# Content evaluation of the inclusive eHealth guide: how to develop interventions for people with a lower socioeconomic position?

**DOI:** 10.3389/fdgth.2025.1528860

**Published:** 2025-10-01

**Authors:** Isra Al-Dhahir, Linda D. Breeman, Jasper S. Faber, Jobke Wentzel, Rita J. G. van den Berg-Emons, Jos J. Kraal, Veronica R. Janssen, Roderik A. Kraaijenhagen, Valentijn T. Visch, Niels H. Chavannes, Andrea W. M. Evers

**Affiliations:** ^1^ Faculty of Social and Behavioral Sciences, Leiden University, Leiden, Netherlands; ^2^ Faculty of Industrial Design Engineering, Delft University of Technology, Delft, Netherlands; ^3^ Department of Health Care and Social Work, University of Applied Sciences Windesheim, Zwolle, Netherlands; ^4^ Department of Rehabilitation Medicine, Erasmus MC, University Medical Center Rotterdam, Rotterdam, Netherlands; ^5^ Capri Cardiac Rehabilitation, Rotterdam, Leiden, Netherlands; ^6^ Department of Cardiology, Leiden University Medical Center, Leiden, Netherlands; ^7^ Vital10, Amsterdam, Netherlands; ^8^ NDDO Institute for Prevention and Early Diagnostics (NIPED), Amsterdam, Netherlands; ^9^ Department of Public Health and Primary Care, Leiden University Medical Centre, Leiden, Netherlands; ^10^ National EHealth Living Lab, Leiden University Medical Centre, Netherlands; ^11^ Medical Delta, Leiden University, Delft University of Technology, Erasmus University, Delft, Netherlands

**Keywords:** health disparities, digital divide, digital literacy, eHealth interventions, inclusive eHealth guide (IeG), socioeconomic position, iterative evaluation

## Abstract

**Objectives:**

eHealth interventions favor those with higher socio-economic positions (SEPs). This can widen disparities, as people with lower SEPs may lack resources and face digital or financial barriers, making tailored solutions necessary. This study evaluates professionals' perceptions of the Inclusive eHealth Guide (IeG) regarding its content. The aim was to ensure it meets the needs of professionals and the targeted lower SEP demographic, thereby enhancing the effectiveness of eHealth interventions.

**Methods:**

This mixed-method study used qualitative research through semi-structured interviews and the think-aloud method with 13 professionals involved in eight different eHealth lifestyle interventions using the eHealth guide. Quantitative feedback was obtained through a survey with evaluative multiple-choice questions. Participants evaluated the IeG at various stages. They identified positive aspects and points for improvement, and provided recommendations for the guide's content and structure.

**Results:**

Participants valued the IeG's practicality and comprehensiveness, noting its usefulness in developing accessible eHealth solutions for populations with lower SEP. They suggested improving content clarity, expanding informational depth, and refining the guide's structure.

**Conclusions:**

The IeG has potential as a valuable tool for professionals developing eHealth interventions for lower SEP populations. Continuous refinement is crucial to ensure the guide remains relevant and effective, contributing to reducing health disparities.

## 1 Introduction

The benefits of eHealth interventions are widely acknowledged. They empower individuals by engaging them in healthy lifestyle activities and self-managing chronic illnesses. This enhances health outcomes, and alleviates the burden on healthcare providers (1–3). eHealth encompasses a range of devices and communication tools, from smartphones and wearables to email and text messaging (4). These technologies play a crucial role in health interventions across platforms, websites, and apps, including smoking cessation and managing conditions like diabetes and cardiovascular disease (5). Technology can benefit individuals through improving adherence and effectiveness of interventions (6). However, achieving these benefits depends on the quality—such as the usefulness and usability—of the eHealth technology. Structural barriers to eHealth use are especially significant for people in lower socioeconomic positions (SEPs), who are disproportionately affected by health problems. These individuals often face systemic exclusion from digital health solutions due to factors such as limited financial resources, elevated stress, and lower digital literacy (5, 7, 8). Consequently, their use of eHealth interventions is often lower, which may further exacerbate existing health disparities (8). Lower SEP is a multifaceted concept encompassing various domains such as education, income, occupation, and neighborhood. It affects a significant portion of the population, with 22% of the EU population or 95.3 million individuals in 2022 falling into this category (9). Variations exist across countries, with areas with a higher concentration of lower SEP people also reporting higher rates of cardiovascular disease and mortality (10). The association between lower SEP and poorer health outcomes and health behaviors—such as smoking and unhealthy diets—is shaped largely by systemic, economic, and environmental inequalities. It is not solely the result of individual choices (7, 11, 12).

However, eHealth interventions hold promise in influencing health attitudes and behaviors among people from disadvantaged backgrounds (13). These interventions can achieve this by incorporating appropriate behavior change techniques and by providing accessible information through features like simple language, visuals, animations, and audio (14). Furthermore, eHealth interventions can significantly improve access to healthcare services by overcoming geographical barriers and associated costs, particularly for working people with limited flexibility for appointments (15). By tailoring interventions to individual circumstances, such as economic and cultural backgrounds, these tools have the potential to empower users to make informed health decisions (16, 17), including users with lower SEP.

To realize this potential, the active involvement of professionals—such as researchers, developers, healthcare providers, and policy officers—is essential. These professionals are responsible for the development, implementation, and evaluation of eHealth interventions. However, they frequently encounter substantial challenges in effectively reaching and engaging lower SEP populations, due to limited practical resources, frameworks, and an insufficient understanding of the lived realities faced by these target groups (18, 19). Without sufficient practical guidance and inclusive design tools, professionals may inadvertently create eHealth solutions that perpetuate rather than reduce existing inequalities (11, 19). People with a lower SEP face not only financial barriers, but also daily pressures such as caregiving, unstable work, and bureaucratic hurdles. These stressors can tax cognitive and emotional resources like attention and self-regulation, creating a scarcity mindset that hinders engagement with eHealth (20). Efforts to promote healthier lifestyles through eHealth must therefore move beyond content improvements and embrace a comprehensive approach that recognizes and addresses the broader challenges faced by this population (11).

A promising strategy to ensure usefulness and usability of the technology is to integrate user needs into the complete developmental process. This is a key element of the Human-Centered Design (HCD) approach, which prioritizes the human perspective throughout the design process (21, 22). HCD emphasizes stakeholder (e.g., users of the technology, healthcare professionals who provide the technology) involvement, to assess their needs, wishes, capabilities, and context, thereby developing eHealth solutions that genuinely align with users' requirements for adoption and sustained utilization. This iterative design process involves early and ongoing engagement with stakeholders, feedback incorporation, and continuous field testing (22, 23). This approach successfully integrates both scientific and practical knowledge, thereby enhancing the user-friendliness and customization of eHealth solutions to adequately meet end-users' challenges, ultimately supporting their effectiveness and impact. Despite these advantages, continuous testing and evaluation with stakeholders is often limited. Constraints include time, funding, limited experience with participatory methods, lack of trust between developers and users, and uncertainty about how to engage end-users meaningfully. These factors result in suboptimal design and implementation of eHealth interventions and resulting in their underutilization (19, 24).

### 1.1 Evaluating the inclusive eHealth guide

To support professionals (e.g., eHealth developers, researchers, health care providers, and policy makers) in designing inclusive eHealth interventions for populations with a lower SEP, we developed the Inclusive eHealth Guide (IeG), a web-based tool grounded in HCD principles. The guide's design was investigated in our prior research (18). The guide was iteratively developed using feedback from professionals across disciplines, ensuring that its content is practically relevant and applicable in real-world settings, providing a comprehensive framework for creating impactful eHealth solutions.

**Figure 1:**
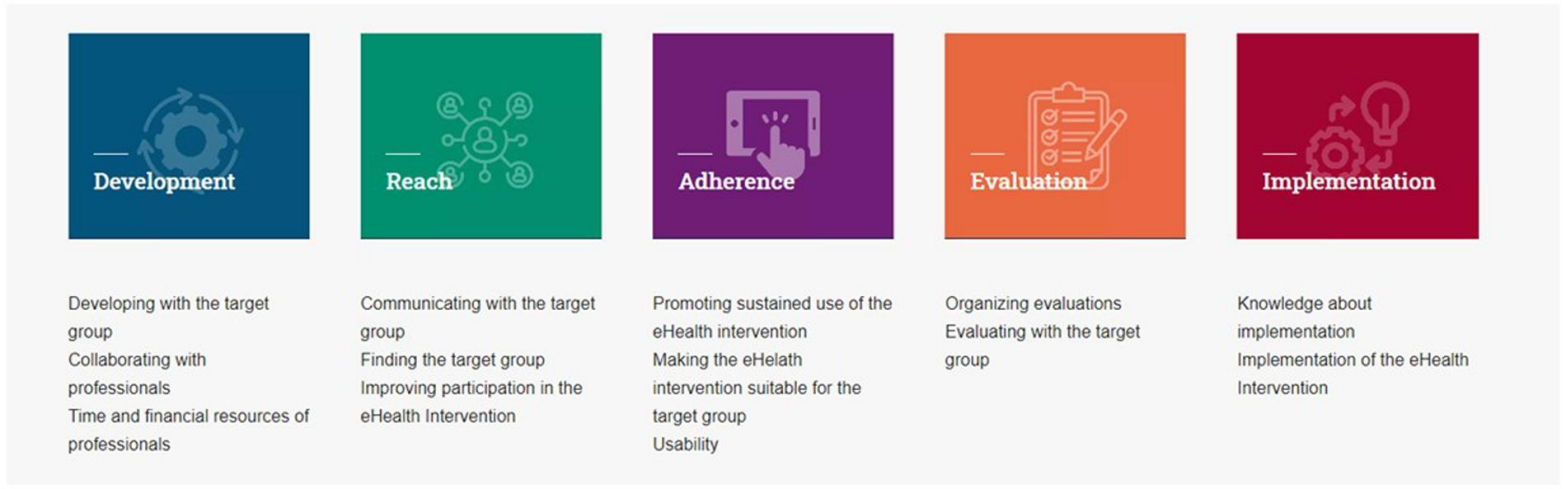
Different stages of the inclusive eHealth guide. Adapted with permission from “Inclusive eHealth Guide”.

**Figure 2:**
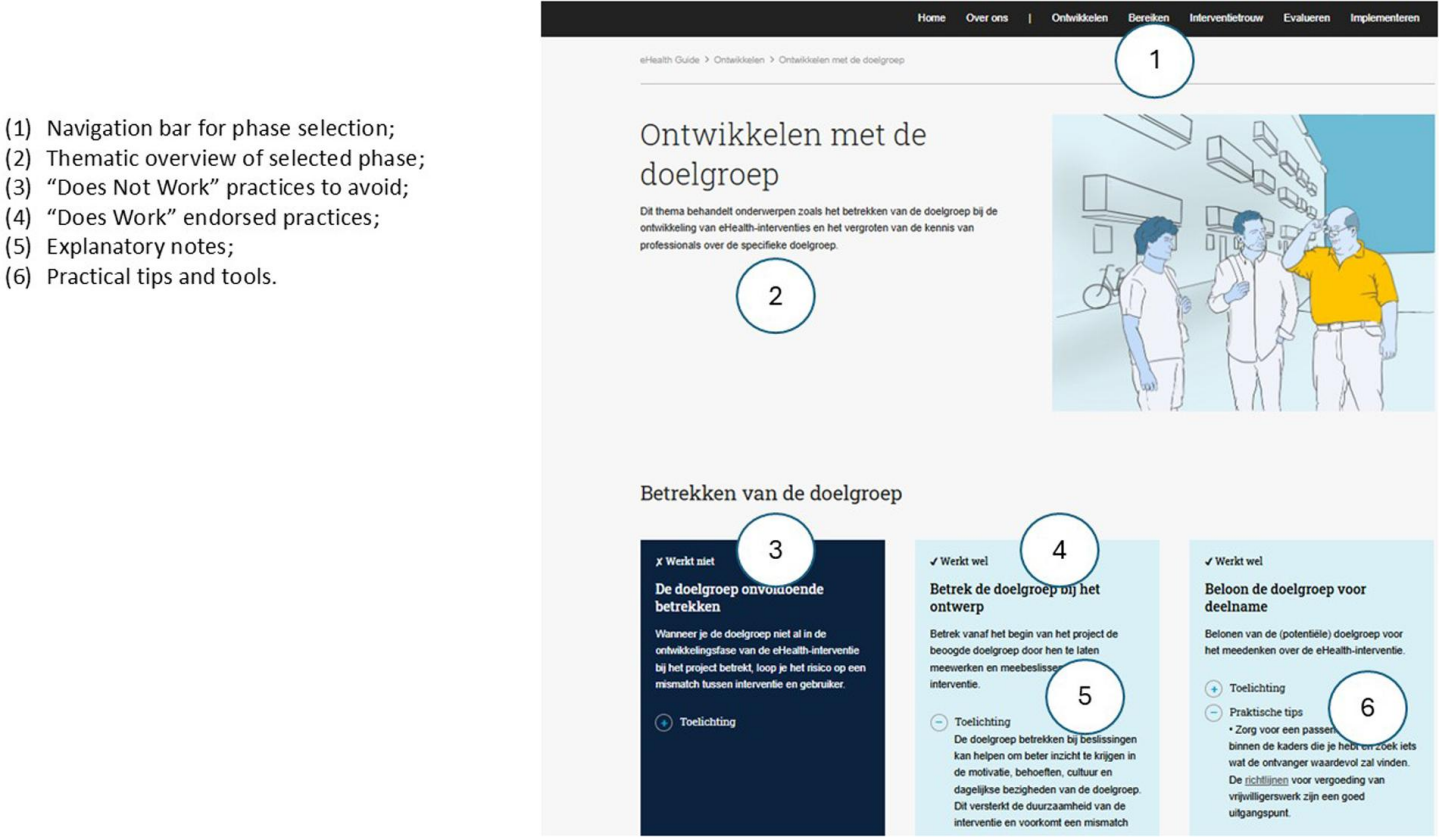
Example page layout from the inclusive eHealth guide, illustrating structure and key features. Screenshot from: “Developing with the target group, Inclusive eHealth Guide.”

The IeG is structured around five key phases of eHealth intervention development: development, reach, adherence, evaluation, and implementation (see Figure 1). Its content is based on previous scoping reviews and empirical studies involving both professionals and people with lower SEP. Each phase addresses common barriers and links them to practical facilitators and actionable recommendations. These are enriched with user portraits, real-world examples, tips, and links to tools and resources [See also Figure 2, elements (5) and (6)]. A visual navigation structure on the homepage allows users to explore the guide flexibly, depending on their needs and the specific phase of their project. An illustration of the IeG is presented in Figures 1, 2, and a detailed description of the guide is included in Supplementary Material S1.

To further develop the IeG, we gathered feedback from professionals to explore how they perceive and apply the guide in practice. Understanding these perceptions is crucial to ensure that the guide aligns with practical needs and effectively supports the implementation of eHealth solutions. This feedback helps identify strengths and areas for improvement, guiding the ongoing refinement of the guide. In doing so, we aim to ensure that the IeG continues to meet the evolving needs of professionals and the lower SEP population, thereby enhancing the effectiveness of inclusive eHealth interventions.

## 2 Methods

### 2.1 Study design

**Figure 3:**
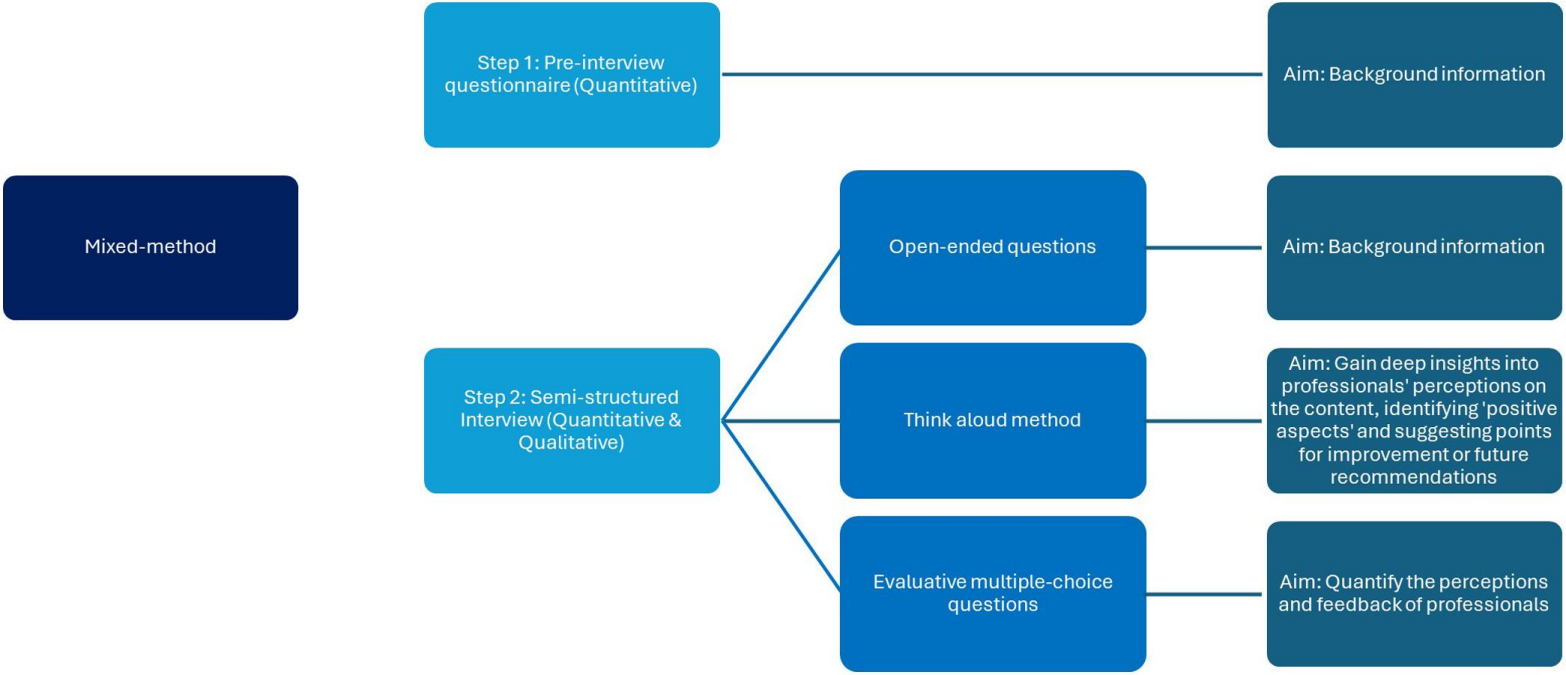
Study design and interview process overview.

Ethical approval was obtained from the Psychology Research Ethics Committee at the University of Leiden (Breeman, dr. L.D.-V2-4262). The IeG was assessed using a mixed-methods framework, blending qualitative and quantitative techniques (see Figure 3). Data were collected in one session using semi-structured interviews and the think-aloud method (25, 26). The think-aloud approach enabled participants to verbalize their thoughts and reasoning, offering rich insights into professional perceptions of the IeG, identifying both positive aspects and points for improvement. Quantitative feedback was gathered through a survey featuring evaluative multiple-choice questions, providing a balanced view of professional responses across different phases of eHealth intervention: development, reach, adherence, evaluation, and implementation.

### 2.2 Ehealth interventions and participants recruitment

**Figure 4:**
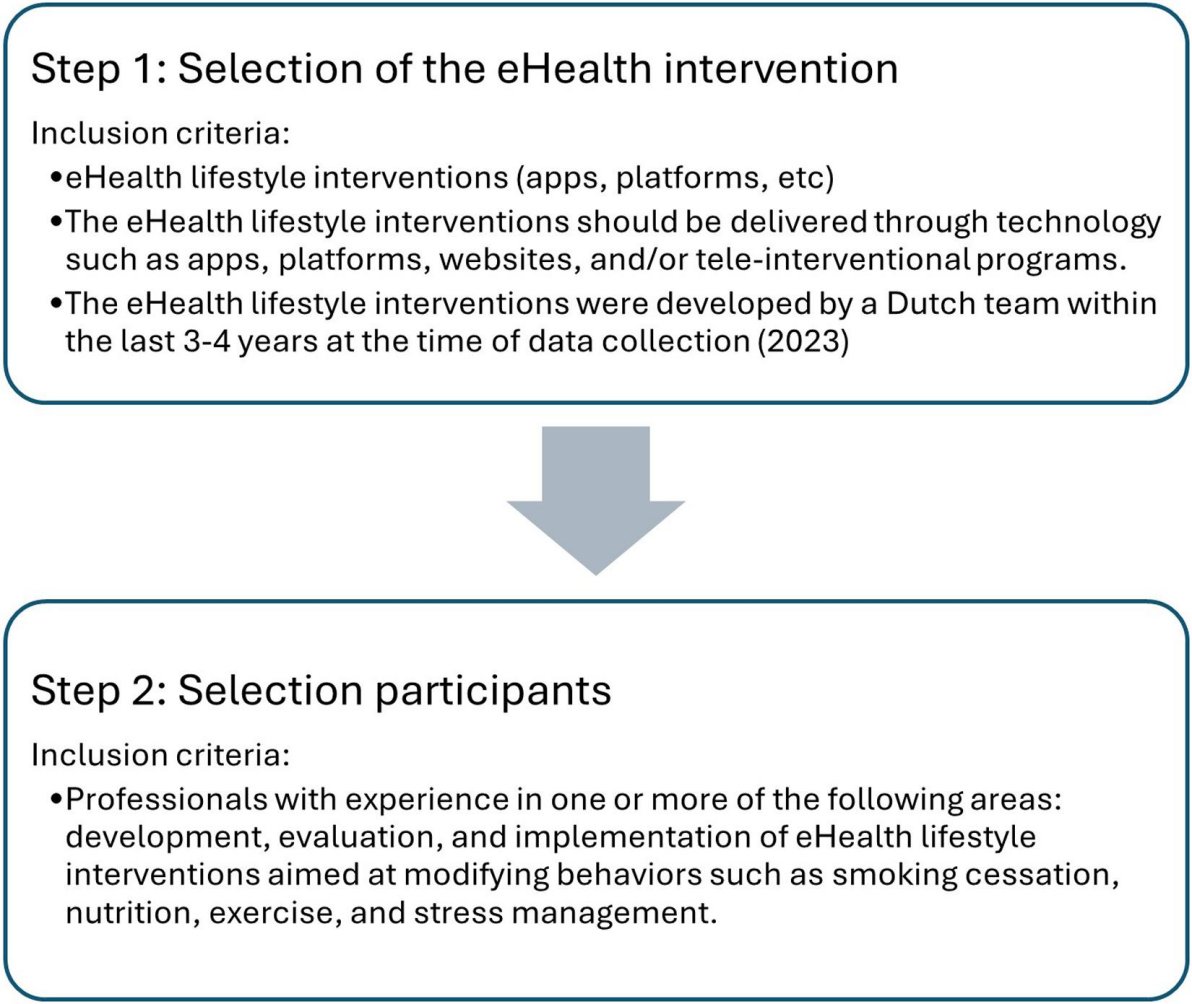
Stages of eHealth intervention and participant selection process.

The relevance of IeG content was evaluated through interviews with researchers and developers from various eHealth-interventions*.* Data was collected in two steps. First, 8 eHealth lifestyle interventions were selected based on specific criteria (Figure 4) to assess IeG practicality and feasibility. This offered the opportunity to identify areas for improvement to enhance IeG applicability. To find suitable eHealth interventions, we compiled a list of eHealth lifestyle interventions based on internet searches and recommendations from colleagues in the field. Subsequently, we contacted individuals responsible for these interventions, inquiring about case suitability based on the selection criteria. In the second step, upon identifying a suitable case, we inquired via email or face-to-face about individuals' intent to participate in this study. Suitable candidates were those involved in at least one phase of their eHealth intervention, excluding those previously engaged in the IeG development. We did not consider participants' experience with lower SEP groups as a selection criterion, because this study aimed to gather diverse perspectives to identify potential gaps in the IeG and improve its general applicability. Including professionals with varying levels of experience allowed us to explore a range of needs and perceptions. This included both those highly familiar with the target group and those less experienced but still involved in relevant intervention work. This variation provided insight into how the guide is understood and used by professionals across different backgrounds and levels of familiarity with inclusive design. The content of the guide was informed by earlier research involving the target group (27). In this phase, the focus was on professionals, as the IeG is intended to support them in designing inclusive eHealth interventions.

Semi-structured interviews with researchers, eHealth developers, healthcare professionals, and other experts involved in these interventions were conducted. Feedback from this range of professionals yielded an overall assessment of the guide's applicability based on their experiences and needs. We interviewed 1–3 individuals from each selected intervention, totaling 13 interviews. This purposive sampling approach ensured the inclusion of diverse professional roles and experiences across different phases of eHealth intervention development, allowing for a broad and informative exploration of the guide's applicability The sample size was considered sufficient to reflect a diversity of perspectives aligned with the study's objectives (28).

### 2.3 Procedure and materials

Interviews were conducted by the first author online or in-person, lasting 50–60 min, and recorded using Microsoft Teams or a voice recorder. The interview process was divided into three segments (see Figure 3). Before the interview, participants received an email with an information letter, a background questionnaire link, and access to the IeG. This pre-interview questionnaire collected data on their roles and experiences in eHealth interventions, particularly concerning lower SEP populations. If participants had not completed the questionnaire prior to the interview, it was completed during the session. The interview began by exploring the resources, documents, and guidelines participants used in their eHealth interventions, their experience with designs for people with a lower SEP, and familiarity with inclusive design principles. This was followed by a discussion on their eHealth intervention involvement, including objectives, target demographics, and SEP considerations (29, 30). Subsequently, the “think-aloud” method was employed. Participants were asked to navigate the IeG online, selecting intervention phases relevant to their experiences. A scenario provided a starting point for exploration, which they could adapt to their needs (see Box 1). They were tasked with examining the homepage, sharing thoughts, noting details, and discussing encountered recommendations and practical advice. In the final part, participants answered multiple-choice questions about the guide's clarity, conciseness, and whether the eHealth intervention phases contained adequate information for developing interventions aimed at people with a lower SEP.

BOX 1 **Scenario.**

**Table:** 

Scenario—Researcher (Development)
Situation: You are currently working on developing the [name] intervention for a broader audience, including those with a low socioeconomic position (SEP).
Website: Imagine that there is a website designed to support professionals like you in the development, implementation, and evaluation of your eHealth intervention for individuals with a low socioeconomic position (SEP). This platform aims to help you reach this group more effectively and improve intervention adherence among them.
Action: You are curious about the information available and decide to explore this resource.
Goal Setting: What kind of information are you looking for or are you in need of.

These items were rated on a 5-point Likert scale, from 1 “very negative” to 5 “very positive”. Participants also provided verbal feedback and answered three open-ended questions regarding guide acceptance, inspired by the Appraisal of Guidelines for Research & Evaluation (AGREE) II Instrument (31). The interview protocol is included in Supplementary Material S2. All information was processed anonymously, with identifying information removed before coding and participant identification codes used instead.

### 2.4 Data analysis

We conducted a thematic analysis of transcribed interviews using Atlas.ti software (32), following Braun & Clarke's (2006) open and axial coding procedures (25). This approach facilitated the identification and organization of recurring themes. The first and third authors independently coded all the data, ensuring depth in analysis. In collaborative sessions, initial codes and themes were discussed, refined, and unified by consensus, resulting in two main categories: (1) positive aspects, which identify the strengths of the IeG, and (2) points for improvement, which offering future recommendations for the guide's content and structure. Although the primary focus was on collecting content-related evaluations, we also obtained valuable insights evaluations about its structure, which were included in the results section. In addition to the thematic analysis, we also analyzed the quantitative data, including participant background information and the quantitative evaluations of the IeG, using descriptive statistics in SPSS Statistics 29 (IBM Corp). The small sample size did not permit inferential analysis.

## 3 Results

### 3.1 Participants

This study involved a total of 13 professionals, including eHealth developers, healthcare providers, researchers, and policy officers, who engaged in eight different eHealth lifestyle interventions, varying from lifestyle management to sleep-related interventions. Of the 54% of participants who reported having worked with people with a lower SEP background, 31% reported having only limited experience (Table 1). The average length of time that participants were involved in these interventions was 2.65 years (SD = 1.32).

**Table 1:** Participant characteristics.

Participant	Sex	Role	Lifestyle Research Experience (Years)	eHealth Experience (Years)	Activities	Working with Lower SEP
1	Female	Policy Officer, Project Leader	5	9	Knowledge gathering and dissemination via Health Disparities Knowledge Center	Yes
2	Female	Researcher	7	4	Scientific research and eHealth intervention development or modification	Yes
3	Female	Psychologist	7	7	eHealth intervention development, application, scientific research, and healthcare practice	No
4	Female	Assistant Professor, Psychologist	8	8	eHealth intervention development, application, scientific research, and healthcare practice	Yes
5	Female	Project Leader	0.5	0.5	eHealth intervention development	Little
6	Female	Researcher	5	4	eHealth intervention development	Yes
7	Male	Project Manager	N/A	2	Scientific research, eHealth intervention development and application	Little
8	Female	Researcher, Healthcare Provider	3	3	Scientific research, eHealth intervention development and application	Yes
9	Female	Researcher	15	8	Scientific research, eHealth intervention development and application	Yes
10	Female	Researcher	4	4	Developing or adjusting eHealth interventions, applying interventions to lower SEP individuals, scientific research	Little
11	Female	Researcher	6	3	Scientific research	Little
12	Female	Researcher, Health care provider	3	3	Scientific research	Yes
13	Female	Researcher	1	1	Scientific research	No

(Little: works occasionally with the lower SEP group, Yes: works frequently with the lower SEP group).

**Table 2:** Descriptive statistics of questionnaire responses.

Question	Score, mean (SD)	Positive, *n* (%)	Neutral, *n* (%)	Negative, *n* (%)
Per phase				
Information Content development (*n* = 5)	3 (0.0)	5 (100)	0 (0)	0 (0)
Information content reach (*n* = 5)	2.6 (0.5)	3 (60)	2 (40)	0 (0)
Information content adherence (*n* = 3)	2.3 (1.2)	2 (66.7)	0 (0)	1 (33.3)
Information content evaluation (*n* = 4)	2.8 (0.5)	3 (75)	1 (25)	0 (0)
Information content implementation (*n* = 4)	2.8 (0.5)	3 (75)	1 (25)	0 (0)
General (*N* = 13)				
Opinions on recommendations (barriers and facilitators)	2.6 (0.5)	8 (61.5)	5 (38.5)	0 (0)
Usefulness of practical information	2.8 (0.5)	10 (76.9)	3 (23.1)	0 (0)
Clarity and conciseness of recommendations	2.5 (0.7)	7 (53.8)	5 (38.5)	1 (7.7)
Information density	2.6 (0.5)	8 (61.5)	5 (38.5)	0 (0)
Practical application	2.7 (0.5)	9 (69.2)	4 (30.8)	0 (0)
Adaptation adequacy	2.7 (0.6)	10 (76.9)	2 (15.4)	1 (7.7)
Evidence-based necessity	2.6 (0.7)	9 (69.2)	3 (23.1)	1 (7.7)

#### 3.1.1 Ehealth experience of participants

The experience of participants in designing eHealth interventions for people with a lower SEP varied. Some directly developed solutions (*n* = 9), while others provided indirect support through consultations or advisory services (*n* = 5). Notably, advisory roles often focused on projects for diverse and potentially marginalized groups facing language and literacy challenges (*n* = 4). One participant designed digital learning interventions outside healthcare to improve access for individuals with limited literacy (*n* = 1). Three participants lacked direct experience in developing eHealth interventions for lower SEP populations.

**Figure 5:**
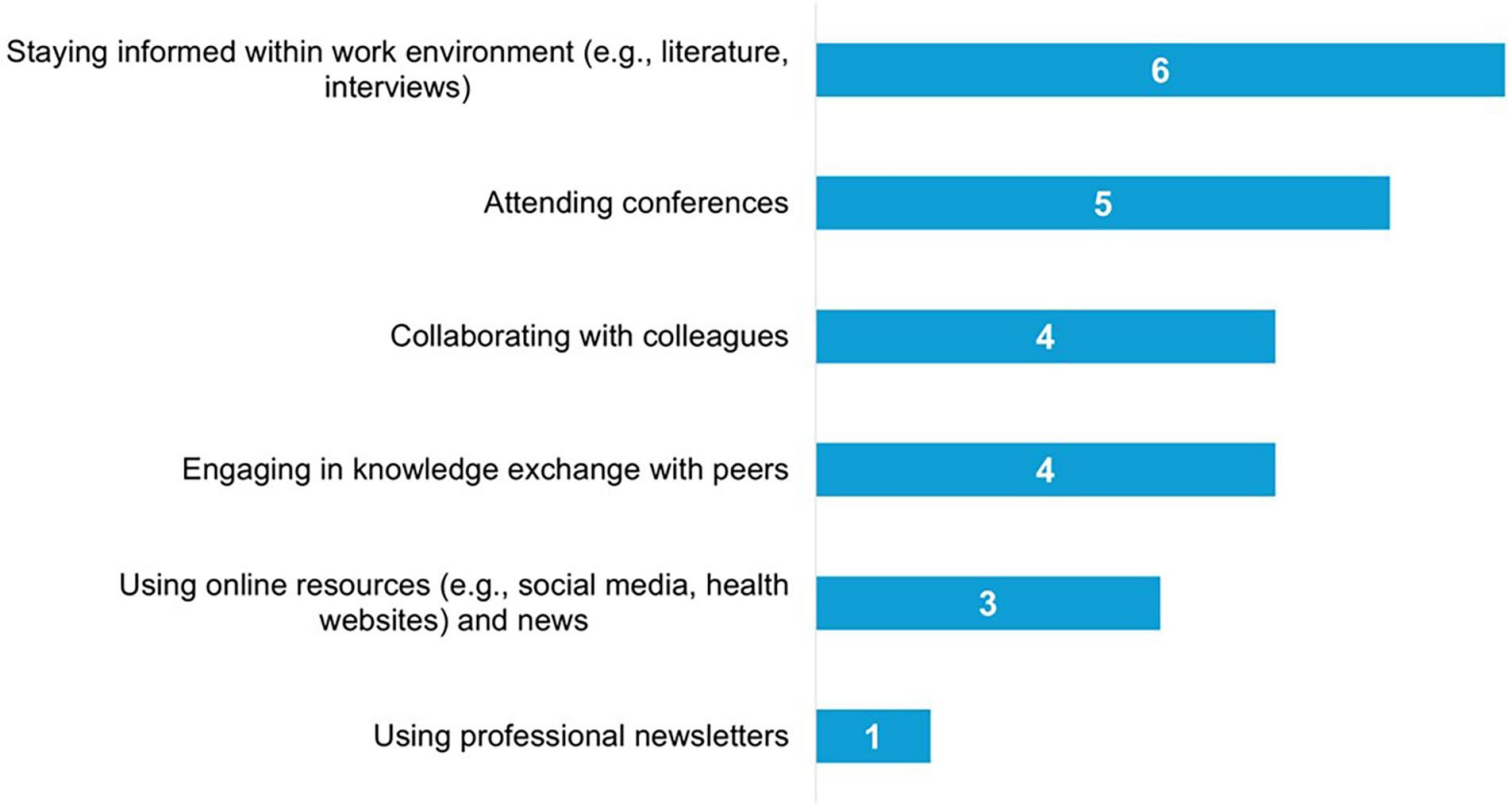
Overview of the sources used by participants to maintain and update their knowledge**.**

Participants reported various methods to maintain and update their knowledge in this area, as derived from the interviews (See Figure 5). Several key resources used for developing eHealth interventions for lower SEP populations included collaborating with the target group (e.g., following the eHealth for All principle), using the Pharos (Dutch Centre of Expertise on Health Disparities) Centre's guidelines, and ensuring B1 level readability, suitable for basic language proficiency.

#### 3.1.2 Ehealth intervention characteristics

The objectives of the eHealth interventions mentioned by the participants included enhancing employee well-being, managing chronic conditions, increasing physical activity, and improving sleep quality. See Supplementary Material S1, Table 1, for more information on the eHealth intervention characteristics Furthermore, inclusivity in eHealth development and evaluation was emphasized as essential for ensuring accessibility across different literacy, digital skill, and cultural levels. This involved simplifying language and using visual or audio elements. A key challenge was balancing simplicity for users with a lower SEP and retaining enough depth for broader audiences. Although this required extra effort, professionals considered it a necessary part of inclusive design.

#### 3.1.3 Suggested users and timing of use

Most participants (84.6%, 11/13) considered the guide valuable for a broad range of professional roles, including developers, researchers, healthcare providers, and project managers. It was perceived as relevant throughout the entire eHealth development process. Participants reported different preferred moments of use depending on their role: developers and project leaders mostly used the guide in the planning or grant-writing phase, while healthcare providers and researchers revisited it during implementation and evaluation. Several participants preferred using it at an early stage, such as during proposal writing or intervention planning, noting it was useful “before you write the grant” (P12). Others highlighted its continued relevance during later phases, such as implementation and evaluation, indicating they would “look at it again” when applying or reviewing content (P2). These findings reflect the guide's flexibility and usability across different professions and project phases. Supplementary Table S2 provides an overview of suggested users and timing of use.

### 3.2 Guide evaluation

Based on their experience with various phases of their own eHealth interventions, participants evaluated the IeG across different phases: “reach” and “implementation” were assessed by 38% of participants (*n* = 5 each), followed by “development” and “evaluation” (31%, *n* = 4 each). The “adherence” phase received less focus (23%, *n* = 3). Table 2 summarizes participants' ratings of the guide across the eHealth intervention phases.

#### 3.2.1 Positive aspects of the guide

Qualitative analysis of the interviews identified 67 themes, including 13 main themes with positive aspects and 19 main themes for improvement or future recommendations. Key themes are presented below, and a comprehensive overview of the themes extracted from the interviews is provided Tables 3, 4.

**Table 3:** Positive aspects, descriptions, codes, and quotes per theme and category.

Theme	Theme mentions (*n*)	Participants (*n*)	Description	Quote
Category: Design and structure				
Information architecture/Navigation	20	9	Clear, user-friendly layout with collapsible sections prevents information overload, enhancing user engagement and content accessibility	*I find it very effective that you can go through briefly with one sentence everywhere and if you want to know more you can press on those pluses.* [P6]
Visual Design and aesthetics	11	8	Attractive design with engaging visuals and a coherent colour scheme enhances the overall user experience and facilitates content	*Yes, I'm a bit drawn to this picture. On the right side. I think, oh interesting, what's it about exactly? Seems like some sort of meeting or something? [P1]*
Presentation “Works” and “Doesn't Work”	8	3	Balances do's and don'ts effectively, providing clear guidance and encouraging best practices in a straightforward manner.	*Yes, I think it's very good indeed to set them against each other like that. From don't do this but do this, so I find it very clear. I give it a thumbs-up I think. Occasionally I found it to be a bit too much text that I think, okay, this could have been summarized a bit shorter and more powerfully. But the format I find quite useful myself.* [P10]
Category: Applicability in various phases				
Development phase	13	4	Useful information on engaging target groups effectively, ensuring technology meets their real needs for inclusivity.	*So involve the target group. If that oh, should I then add technology? Yes, so If I come to this website, then I will delve into it, so This is nice, right? Because I'm already all here. The ins and outs will go, but I can also first look over. Technology, lack of insight into the use of devices by the target group. Yes, I had talked about that myself, right? That I would then search myself.* [P5]
Adherence phase	12	2	Useful information on rewarding participation and enhancing social connections, emphasizing usability and health literacy.	*Yes. But the information itself I think is really, really good. And the tips that are given are all things that I think yes, That's indeed very important to. Take into account and think about, yes, during the development and implementation of such an eHealth intervention so in that respect I think you've made a nice selection of what's available.* [P*10]*
Evaluation phase	8	2	Useful information on creating a positive atmosphere and leveraging expert advice for evidence-based interventions.	*Well, we still have some gains to make. This is already useful information, because we can still use this. Indeed, we have asked for feedback.* [P3]
Implementation phase	6	3	Useful information on effective communication and practical tools for building trust and ensuring successful implementation.	*Yes. Oh yes, good, that there's an evaluation tool included. A link right away. Yes, This is very practical information that is very nice to have, I think.* [P3]
Category: Phase-based content relatability				
Development Phase	8	4	Information is recognized (e.g., with personal insights, utilizing simple evaluation methods for improved outcomes)	*And what works well? Yes, aligning budgets. That's very important, indeed, that lack of insight. I think it's always good that not everyone is doing it for the first time in a team, because then you miss out on a lot of information. [P2]*
Reach Phase	6	2	Information recognition in the reach phase	*Involving the environment in the intervention. … So we have also been at the group consultation indeed for that reason as well. [P8]*
Adherence Phase	7	2	Information is being recognized with one's own project in the adherence phase	*Okay, I'll focus on usability because that was an important part of my dissertation. It showed that indeed, usability is one of the key factors that can influence the effect of eHealth. [P10]*
Evaluation Phase	3	2	Information is being recognized with one's own project in the evaluation phase	*I knew a lot of these things from my research*. [P6]
Implementation Phase	6	4	Information is being recognized with one's own project in the implementation phase	*So I actually think that's good. So yes, this one I completely agree with. Well, that's exactly what we ran into, so it's in the right place. [P7]*
Category: Practicality and Resourcefulness	31	12	Including practical tips and relevant links enhances the website's usefulness for users seeking hands-on information	*I found the scientific literature quite extensive, and the links I saw were interesting. I'll look into that. [P9]*
Category: Value of Practical Tips	22	8	The practical tips were valued by professionals	*And glad that there are many practical tips, because I would primarily use it for that If you want to know. What is the theory about implementation of intervention content?.…So that seems nice and all the links to various useful websites.* [P4]
Category: Conciseness	13	7	The guide offers clear, concise content with practical tips for easy understanding and application	*That's a big plus, I breezed through it. It's all very, very clearly written, yes.* [P2]
Category: Scientific information	6	7	Balancing depth and design, a top resource for comprehensive research references	* And I found the scientific literature to be very extensive and the links I saw in it, I found interesting. Then I'll look into that.* [P8]
Category: Comprehensive Guide	7	3	Detailed resource covering all essential aspects	*In my opinion, is quite complete.* [P4]
Category: Sharing the guide with colleagues	4	2	Valuable collaborative guide, highly recommended for professionals with diverse populations	Yes interesting, I am going to share this with colleagues who do a lot of writing and developing to take a look at it when we develop something again. [P5]
Category: Addressing overlooked topics	2	2	Provides valuable insights into often forgotten but important subjects	*Oh yes, privacy …, I think it's good that pieces are always added. I think we often forget about that, don't we? And it is indeed important, yes, I'm fine with that. I think it's still good to make agreements about this together. …* [P7]
Category: Initiative and Importance	2	1	Praised as a vital initiative, the guide fills a significant gap, offering in-depth information valuable even to seasoned experts	*I think it's a very good initiative for starters to do this. I think it's a very important gap to fill and yeah.* [P11]

Theme mentions (*n*) = total number of times the theme was mentioned.

Participants (*n*) = number of unique participants who mentioned the theme*.*

**Table 4:** Recommendations, descriptions, codes, and quotes per theme and category.

Theme	Theme mentions (*n*)	Participants (*n*)	Description	Quote
Category: Clarity and Accessibility of Content				
Increase clarity	15	6	Make the content on the website more understandable and direct	*Or well, I know what ‘ ISO’ is a bit, but I don't quite understand how it fits within the target audience here. But it's mentioned here. Ultimately, it's just there. Okay.* [P7]
Simplify Terminology and Jargon	10	7	Revise technical terms and jargon within the Guide	*Yes, I don't exactly know what you mean by the participant panel actually. And I think that's because a large part of my PhD does not involve implementing eHealth interventions. I have worked more, just more as a doctor, but not all my. All those terms are known to me, probably the people who no longer work would know, but maybe you could just paste a link underneath with terms that are assumed to be known. But that you just put in, a sentence that shows what* it means when you click on it. [P12]
Balance repetition	10	7	Achieve an optimal balance in repeating content for emphasis, while avoiding redundancy	*Yeah. So, I really like the second point and not so much the first point that doesn't need to be repeated.* [P11]
Typo error	9	6	Identifying and correcting typographical errors	*Participants reads text aloud: “Doesn't work, mismatch between evaluation method and the target group, I think” (sentence missing on the website).* [P3]
Value of Explanatory Sections	9	4	Ensure explanatory sections add significant value and clarity	*Yes, every time with these explanations, I think, yes, why does it need to be here and can't it be summarized more briefly in a piece of text? It would save less clicking and expanding.* [P10]
Open doors	5	1	Begin with simple, straightforward information that gradually leads users to a deeper understanding	*Looking at “Lack of Financial Resources”: Yes, it feels a bit like an open door to me, but I think this is a super interesting and important aspect, as I think many people encounter this.* [P7]
Conciseness in Recommendations	5	2	Focus on delivering succinct and clear recommendations	*Occasionally, I found it to be a bit too much text, thinking, okay, this could have been summarized more concisely and powerfully. But I find the format very useful myself.* [P10]
Desire for summarized content	9	1	Introduce brief, clear summaries of essential information, enabling users to quickly grasp critical points without needing visiting external resources	*… it still requires a lot of research on my part. What I find difficult, I would prefer to have it more targeted and concrete directly on the website itself, yes.* [P10]
Category: Depth and Practicality of Content				
Direct Guidance	32	10	Create content that offers explicit, actionable advice.	*… but with some practical tips I thought. It's more of an explanation than a tip, so to speak. In my mind, a practical tip is something that I can immediately do*…[P3]
Enhancing Practicality	25	9	Provide concrete examples and tools for immediate application	*Yes, and I would maybe want something more, often with peer groups, maybe an example of where that has been done and how it was done.* [P8]
Desire for Detailed Information	13	8	Provide detailed information	*And then I guess, yeah, like literally, what should I do to make it easier for people.* [P11]
Quantity of Practical Tips	13	8	Find a balance between the quantity of tips and conciseness	*“I think the information contained is good, but as I've mentioned, I found some sections quite long which makes me think that the information might not be fully absorbed, but the things that are there are relevant because I'm familiar with them.”* [P7]
Useful Information and Resources	5	2	Provide essential and accessible resources	*“*…*I'm not sure if it's there, but I found ABC1.nl very useful.”* [P6]
Different Content Needs/Content Relevance	2	2	Tailor content to diverse user needs for relevance	*Well, fine, time and financial resources. Okay. This is perhaps something that is less relevant for me, you know. I think this is before people start developing things at an agency. (P5 means “financial resources”)* [P5]
Category: References in the guide				
Balancing practicality and scientific rigor	6	4	Incorporate practical examples and direct resource links	*Yes, for instance, here is information about training and courses to gain skills. The previous one had an example of an instrument immediately. Perhaps it would also be nice to have a practical example of a useful training, for instance.* [P1]
Consistent References in the Guide	2	2	Ensure consistent and evidence-based references	*Yes, and every time I expand the explanation and read it, maybe also because I have a scientific background, but then I immediately think of. Oh, what are the sources of this then?* [P4]
Providing sources	4	3	Incorporate practical tips with direct links to sources for depth	*I like that you have, uh, references. For us, that's very important for researchers, and maybe you could hide them like behind numbers or something. So it doesn't take up so much space.* [P11]
Category: Lay-out				
”Works Well” vs. “Doesn't Work” presentation	29	10	Balancing positive and negative feedback for clearer guidance	*Well oh, This is also clear, those blocks. What works, what doesn't? I don't know if I would do what doesn't work first and what works well after …* [P2]
Content categorization	20	7	Effectively organizing information for immediate and relevant access	*Yes, every time here with that explanation, then I think, yes, why does it have to stand here and can't it be summarized shorter in a piece of text? It saves less clicking and unfolding work.* [P10]
Visual Communication and Design	17	7	Using visual elements to enhance comprehension and appeal	*Well, “doesn't work,” I do associate with red, perhaps.* [P5]
Clarity and structure of information	8	4	Streamlining content for quick and easy comprehension	*Yes, I might have expected, perhaps, some sort of bullet points here. So that you can quickly see which social media channels they use. Or if they're not reached through social media at all? Or the consultation room, something like that. Maybe it gets to the point faster.* [P5]
Phase categorization	12	4	Clarifying project stages for focused development and implementation	*Yes, because But that, that's also a bit like always reaching then not under the part of implementation?* [P8]
Interface usability	5	3	Optimizing navigation and interaction for user-friendly experiences	*Maybe I don't find it critical, But it's quite long scrolling down those texts so Maybe If it could be a bit smoother or in a kind of small summary that you could click through, but then there would need to be more texts than now.* [P7]
Category: Hierarchy/Priority	5	2	Prioritize essential actions	*Oh, my …, I really don't have time for this, so to say. Yes, but if you want to know more, you can expand the explanation and if you want to know even more, you can expand the practical tips too. I find those sometimes a bit long.* [P6]

Theme mentions (*n*) = total number of times the theme was mentioned.

Participants (*n*) = number of unique participants who mentioned the theme.

Feedback was largely positive across all phases: development (5/5 positive), reach (3/5 positive), adherence (2/3 positive), implementation (3/4 positive), and evaluation (3/4 positive). Furthermore, 69.2% (9/13) reported the recommendations as sufficiently informative for independent progress, while 76.9% (10/13) confirmed the guide's adequacy for tailoring eHealth interventions to a lower SEP audience. Tables 3, 4 present examples of participant quotes highlighting the positive aspects of the IeG, as well as recommendations for its improvement.

##### 3.2.1.1 Content of the guide

Participants found the guide's practical information useful (76.9%, 10/13 positive) and generally recommendations also clear (53.8%, 7/13 positive). The density of information within the recommendations was also positively viewed (61.5%, 8/13 positive). More than half of the participants (7/13) valued the guide *concise clarity*, appreciating the brevity and straightforward presentation that facilitated quick and easy understanding. One participant (P2, Researcher) noted that “everything is nicely clear, and it is also nice that you can click through,” highlighting the usability of the structure.

Reflecting on the *IeG applicability and practicality*, almost all participants (12/13) across various phases concurred on several positive aspects. The majority (8/12) valued the website's practical tips, including the concrete guidance, tools, and strategies provided. They appreciated the easy access to information via evaluation instruments, participatory research, and hyperlinks, finding it helpful for implementing interventions and overcoming challenges. As expressed by one participant (P9, Researcher), the guide is “filled with concrete tips that are feasible and understandable”.

Furthermore, several participants (*n* = 4) emphasized that the guide's principles and practical recommendations are applicable beyond digital health interventions or lower SEP groups. For instance, one participant noted that “most of the tips can also be used for non-digital interventions” (P3), and others highlighted its potential value for broader intervention design in areas such as lifestyle support and rare diseases like Huntington's (P4).

In the *development phase* all participants (4/4) appreciated the focus on understanding the target audience's technology usage. Practical tips like participatory design techniques and community engagement were seen as instrumental for developers. One interviewee (P4, Researcher) remarked, “This is enjoyable, all these concrete details. Yes, I find that very useful”.

In the *adherence phase*, participants (2/3) valued the depth and relevance of information. Tips on rewarding participants and setting achievable goals were recognized as crucial for enhancing user engagement and adherence. This appreciation for the guide's practical examples was expressed by one psychologist (P3), who noted: “Good to see there are many cases and examples included. If you need more information, you can read further”.

In the *evaluation phase*, half of the participants (2/4) valued content practicality, focusing on positivity and convenient processes. Community-based participatory research, expert advice, and stakeholder involvement were believed beneficial. Innovative, inclusive methods for lower SEP populations were also valued. Finally, in the *implementation phase*, the majority (3/5) found the information directly beneficial for developing intervention strategies, with concrete examples emphasizing the importance of thorough preparation.

##### 3.2.1.2 Design and structure of the guide

Participants expressed positive feedback regarding the website's design and structure. The majority (9/13) found the *content presentation* to be concise and user-friendly, with the option to delve deeper into the material through additional links without feeling overwhelmed by all information. One researcher highlighted that “you can go through briefly with one sentence. and if you want to know more, you can press on those pluses” (P4), referring to the expandable sections on the website that offer more detailed information.

The recommendations “Works” and “Doesn't Work” were well-received (61.5%, 8/13 positive). Participants (3/13) perceived the “*Works” and “Doesn't Work” presentation* format as insightful, promoting a deeper understanding of best practices by showcasing successful strategies alongside common pitfalls [see Figure 2, elements (3) and (4)]. The sequential flow of content, transitioning from problem identification to solutions, was considered logical and beneficial for learning and decision-making. The website's *aesthetics* also received positive remarks, with participants (8/13) praising the clarity of the images, color coding between “Works” and “Doesn't Work” sections, and overall layout.

The necessity for recommendations to be based on scientific evidence was affirmed by 69.2% (9/13 positive). Some participants (4/13) underscored the value of grounding website content in *scientific* information, such as literature and references, to demonstrate credibility and appeal to academically inclined users (i.e., highlighting the importance of evidence-based and experience-based input). Participants appreciated the inclusion of scientific sources to underpin content validity and reliability. They also preferred practical applications, suggesting a balance between academic rigor and real-world relevance.

#### 3.2.2 Recommendations for the guide

##### 3.2.2.1 Content of the guide

A clear theme emerged around the need for improving the *clarity and accessibility of the guide's content*. The majority of participants (7/13) underscored the importance of *simplifying terminology and reducing jargon* to make the content more approachable. This included suggestions to provide clearer explanations and definitions, potentially through hyperlinked terms, to aid in comprehension. One participant (P4, Researcher) noted, “Evaluation—what kind of evaluation is that, because of course, you can evaluate during the development phase,” illustrating the need for more precise terms.

Additionally, some participants (5/7) recommended a nuanced view on repetition; while it can reinforce key points and aid retention, excessive repetition was seen as counterproductive. This was reflected in the question by a policy officer and project leader (P1): “Which professionals are being referred to here? Are they healthcare professionals?”, indicating a need for clarification and consistency.

All participants indicated a preference for more *in-depth and actionable content*. This included requests for step-by-step instructions on specific processes (e.g., collaboration strategies, team communication), concrete examples, case studies, and links to successful projects for enhanced learning and application. A researcher (P10) remarked, “What are those rules of thumb that I need to take into account?”, emphasizing the desire for practical, directly applicable information. However, some participants (3/13) expressed a desire for concise information delivery to avoid overwhelming readers.

###### 3.2.2.1.1 Expanding information content

The participants offered *broad suggestions* for enriching the guide's informational content that are applicable to various phases of the guide (see Table 5). The incorporation of both positive and negative examples was suggested to facilitate comprehensive learning, reflecting on past successes and failures. Participants valued insights from other projects' experiences, recommending the sharing of best practices and lessons learned. The balance between acknowledging participation and avoiding undue influence was noted, with suggestions for small incentives like supermarket vouchers to maintain engagement. Additionally, guidance on managing privacy concerns in different project phases was sought, including simplifying complex privacy regulations and effectively communicating them to end-users.

**Table 5:** Recommendations on expanding information content, descriptions, codes, and quotes per theme and category.

Theme	Theme mentions (*n*)	Participants (*n*)	Description	Quote
Category: Expanding information content guide: general				
Privacy Regulations	5	4	Navigating General Data Protection Regulation complexities during all project phase	*Or at least, and also look, we are naturally quite involved with privacy from our regular work, but I can imagine that if you are, for example, just a community worker, you might be less aware of how quickly you actually deal with data processing and what you do with it. So I think it is good to be aware of that.* [P7]
Funding and Sustainability	7	3	Strategies for financial support and enduring project viability	*Yes, contracts with health insurers. That could help a lot if you have an eHealth platform, we find. We tried it for another app, but we find it quite complicated to host the app ourselves and then get it covered by a health insurer. I am curious if there are good examples of eHealth platforms and which health insurers participate in them, so that you get a bit of a head start in understanding what is and isn't promising…*[P2]
Extent of Changes	2	1	Focus on the scope and impact of potential changes is critical	*So, yes. And, of course, it also varies depending on the type of project or whether it's within a research grant or not.* [P4]
Suitable Rewards	5	5	Establishing appropriate incentives and recognitions.	*Here, I think you can sometimes reward people too much. In the sense that if you suddenly give them a €100 voucher or something, they might say, ‘Yes, but I wasn't doing it just for the money,’ and then they feel not taken seriously anymore, so it needs to be appropriate.* [P4]
Category: Expanding Information Content Guide: Development				
Finding the target group	1	1	Strategies for identifying and reaching the target audience	*Yes, what I initially miss a bit here is, where do I find my target group? So maybe there should be some sort of analysis. Are they on Facebook, or do you have to find them with posters at the general practitioner's office? I can imagine. Maybe it's mentioned somewhere, but that would be. That's knowledge I find interesting when I work for clients like FMS or NFU?* [P5]
Organizing focus groups	1	1	Challenges and tips for efficiently organizing focus groups	*No, so for example, because the next part does indeed concern time and resources. If you want to organize a focus group, with professionals, that is quite a challenge. But maybe it's mentioned here somewhere? Something like that would still be relevant, yes. Because they have so little time, how do you get them to the table, so to speak. But really super good tips.* [P3]
Category: Expanding Information Content Guide: Reach				
Collaboration Complexity	3	1	Address challenges in multidisciplinary collaboration for project execution	*Yes, this is also quite difficult, because yes, of course, it's super good to work together with different disciplines and professionals, … then you still have to get that together completely, so that's easier said than done in my experience.* [P10]
Diverse Communication Strategies	4	1	Employ multimedia approaches for broader and more inclusive outreach	*Yeah. It should highlight that if you're committing to an international app, then also be prepared to be called in Turkish. And so I should be able to answer the phone.* [P11]
Audience Engagement Strategies	7	5	Identify and utilize preferred communication channels for effective outreach	*Like, that you quickly see which social media channels they use. Or if they are not reached* via *social media at all? Or the consultation room, something like that.* [P5]
Social involvement	5	3	Consider family support for patient autonomy and app utilization enhancement	*…It indeed can help with them feeling supported, just also in the intervention or something, so I think that in itself is still.* [P9]
Testing Approach	1	1	Strategies for effective target group testing to refine project outcomes	*And then it mentions testing with the target group itself here. Yes, okay But how should I approach that then?* [P10]
User Adoption Challenge	1	1	Overcoming initial disinterest in app adoption through tailored engagement	*Is it not that they don't necessarily have the need for an app initially and maybe if they work with it they might discover oh, this is actually very nice and can help, but it's just not the first thing they think of.* [P9]
Category: Expanding Information Content Guide: Adherence				
Enhanced Personalized Engagement	8	3	Tailoring feedback to boost motivation and maintain engagement.	*But maybe something more personal as motivation like “good job”. And that's what I did with the [name of the intervention]. It was simply: “Okay, these are the tasks. This is the information you need to read. … P13:” “Yes, there's a lot of resistance, and as soon as you're even a little nice to them, doing well, they quickly find it too patronizing. So, it's difficult, right? A tough balance.* [P13]
Involving Social Support	3	1	Engaging participants' social circles to enhance intervention outcomes.	*…You're already asking something from people themselves, but then also from their environment. I do know it works, though. It definitely helps.* [P13]
Use of Gamification	3	2	Simplifying gamification to ensure accessibility and enjoyment for all	*For anyone it should be a very simple, intuitive, relaxing game instead of something and another chore.* [P11]
Visual Design and Technical Support	2	1	Focus on appealing design and user-friendly technical aspects	*Well, actually no. Maybe, because it's technical support, more about what does it look visually? I might have expected to get tips on how to design and develop it, what to consider. I wouldn't necessarily know that now, suppose I had to develop it myself.* [P13]
Reminders	2	1	Use active, situation-aware reminders to enhance engagement	*Out my experience is that if you let an intervention, an online program or platform, send reminders itself, usually at fixed moments, this can work very much against you.* [P13]
Concerns about Health and Digital Literacy	1	1	Address health and digital skills during development	*Yes, and what I then also wonder, when I read all this about health skills and digital skills. Those are also things that you then already need to take into account during development, it seems to me. So, I hope that this has already been addressed.* [P10]
Encouragement over Punishment	1	1	Prioritize rewards over penalties for better motivation	*… I just find the other two points, say the first two, they feel less like a reward and more like some kind of punishment if you don't do something* [P10]
Category: Expanding Information Content Guide: Evaluation				
Collaboration and Engagement with Stakeholders	10	4	Emphasize stakeholder engagement	*I notice that especially in a hospital for example where, well, hierarchy is very important and whatever If the main person says no, then it just doesn't happen, even if it's very good* [P6]
Different evaluations	11	2	Clarify types and purposes of evaluations within intervention stages	*Consider setting up an evaluation plan: Maybe it can be split or something. This interim evaluation and the effectiveness evaluation….* [P4]
Evaluation process enhancement	6	3	Detail enhancing evaluation processes with stakeholder and target group input.	*Well, I think maybe I misread it, but I thought that to improve an intervention and thus also adherence, you have to evaluate it. So you could mention that as a negative point. If you don't apply an evaluation, you can't better align the intervention with your target group. If you don't evaluate, not only under your target group but also definitely among professionals, because it also has to be user-friendly. They have to support it.* [P12]
Participant selection and diversity	4	2	Focus on diverse participant selection for comprehensive evaluation feedback.	*Only how do you prevent, that you skip some people in the target group, say. How do you do that then? How do you ensure you're not biased So to work, say. I'd miss a practical tip there.* [P3]
Planning and considerations related to a project	1	1	Incorporate evaluation planning early in project proposals and implementations.	*Actually, I think it's also something like if you're applying for a grant or something where you immediately have to take into account. That you have space in advance for just conducting the evaluation, but also for taking into account the things that come out of it.* [P4]
Category: Expanding Information Content Guide: Implementation				
Anonymity and Privacy	2	1	Ensuring participant data anonymity with no impact on care quality	*Yes, nice. Maybe it's indeed very good to emphasize that the data are anonymous and that it has no consequences for the care you receive, because that's how it is with us.* [P12]
Implementation Support	1	1	Offer practical tips for smooth implementation, like accessible contact options	*Yeah, alright. Yeah, I'm not going to go in there now, but something that maybe you don't have is like practical tips to have the implementation goes smoothly, like having a phone number they can call. We had a lot of trouble with that, that we were only available by email, but a lot of people wanted to call. Maybe under ‘Development’ maybe you have it to have voice recording or like voice typing like Siri option.* [P11]
Implementation Planning and Risks	2	2	Address comprehensive implementation aspects including preparation and execution risks	*This now naturally includes financing, project, preparation, and privacy. I just think that's a bit all-encompassing for implementations, aren't they? Would you add more chapters or something? Of course, implementation doesn't just start with preparation, but ultimately the execution of it as well, wouldn't you maybe want more information about that or something?* [P1]
Legal and ownership considerations in eHealth implementation	2	1	Addressing legal ownership and responsibilities in eHealth projects	I think *that's quite complete, the only thing I can think of is that point about ownership, I think. You really need to involve a lawyer in that. Sometimes I think it depends on how the cooperation is, but you have to lay down something about it. Naturally, that's important.* [P4]
Implementation Focus and Engagement	5	1	Ensuring user and stakeholder engagement in eHealth implementation	*Implementation of eHealth intervention' is also very important here, as those who use it and actually have to implement it or offer it to the patient need to be motivated, involved, and believe in the intervention* [P12]

Theme mentions (*n*) = total number of times the theme was mentioned.

Participants (*n*) = number of unique participants who mentioned the theme.

Specific *recommendations for each phase* were made, such as methods for identifying the target audience, analyzing their engagement, and tips for organizing focus groups in the *development phase* (*N* *=* *2*). Also the importance of dynamic scheduling and incentivizing focus group participation was indicated. As one researcher (P9) explained: “Also, determine what a good moment and time might be, and then, as a researcher, you will need to be flexible. Because it will not always be, say, the typical 9–5”..

In the *reach phase*, some participants (2/5) highlighted the importance of *social involvement* and practical engagement strategies (4/5), suggesting the inclusion of patients' family members in eHealth interventions (e.g., translating, app usage) to enhance audience engagement. However, it was also emphasized that the children's age and the potential burden on them must be considered.

In the *adherence phase* (*N* = 3), suggestions focused on personalized engagement and the use of gamification, recommending customization to reflect individual user needs and achievements to enhance motivation and adherence. One researcher (P11) reflected: “…And we also did the gamification…It shouldn't have been too complicated. I think we made it a bit too complicated”..

In the *implementation phase*, participants (*N* = 5) emphasized the need for thorough planning covering aspects like financing, project preparation, and privacy. Emphasizing both planning and execution, they advised the inclusion of concrete examples to illustrate the impact of inadequate implementation. One participant (P12, Researcher and Healthcare Provider) stressed: “Implementing the eHealth intervention is crucial; those using it and offering it to patients must be motivated, involved, and believe in the intervention”..

They indicated the necessity of *securing structural funding and ownership issues*, including intellectual property rights, to ensure the sustainability and success of eHealth interventions. Additionally, the implementation of practical support mechanisms, such as providing direct contact options (e.g., a dedicated phone line and email support), was recommended to improve accessibility and user support.

##### 3.2.2.2 Design and structure of the guide

Design suggestions were provided by participants. They suggested 13 recommendations for improving the website's layout design. Mixed opinions (9/13) were expressed about the “*Works Well” and “Doesn't Work” sections*, with a preference for direct access to solutions and a need for clearer guidance through a balanced approach to both sections. The importance of logical and intuitive *content organization* was emphasized by 7/13 participants. Additionally, adding visual aids, such as charts, diagrams, and images, was suggested by 7 participants and found to be valuable.

## 4 Discussion

This study aimed to explore professional opinions on the IeG (18), a guide designed to support eHealth intervention development for lower SEP populations. The IeG was positively evaluated across different professional fields, particularly for its practicality, broad applicability, and inclusion of real-life examples and actionable suggestions. According to some professionals, the guide's applicability may extend beyond eHealth, offering support in non-digital settings as well. They cited elements such as participatory design and accessible communication as relevant across various intervention contexts. However, further exploration of this potential is warranted. To further enhance the guide's utility, it is necessary to improve accessibility, enrich content with more in-depth information, refine recommendations for specific developmental stages (adherence, implementation, and evaluation), improve the design for a better user experience, and ensure a balance between scientific depth and practical relevance.

### 4.1 Practicality and relevance of the IeG

The practicality and relevance of the IeG were often highlighted by professionals from different disciplines. They appreciated the guide's practical examples and actionable advice for developing eHealth interventions for people with a lower SEP. Its comprehensive framework cover the full development process, aligning with requirements identified in our previous study (18), and stemming from participatory design approaches (24) and HCD principles (22), focusing on user needs and stakeholder involvement.

Although differences were not systematically explored, professionals with less experience working with lower SEP groups may have underestimated the complexity involved in tailoring eHealth interventions. This potential gap between expectations and the realities of practice could influence perceptions of required time and resources (33).

However, feedback highlighted areas for improvement. Despite positive reception, calls exist for broadening topics and better accessibility through clearer content categorization and providing more detailed implementation guidance with “actionable advice”. Experienced professionals, especially those working with people with a lower SEP, suggest the need for detailed instructions and real-life case studies to navigate unconventional situations. Recommendation reflects a broader agreement on the importance of actionable advice, a critical need identified in our prior study (18). This need has not been fully met because we aimed to keep the content concise, based on the needs of participants from our prior study, and not all information could be supported with practical examples due to the lack of specific information for people with lower SEP. Existing guidelines, such as those from the World Health Organization (WHO) and the National Health Service (NHS), offer some eHealth development direction, focusing on people in vulnerable positions (34–36). However, these often lack the concrete, actionable knowledge—the “how”—professionals need to navigate complex real-world situations. This need for practical, step-by-step guidance was raised by participants in the current study and supported by our previous research that informed the development of the IeG (18). Without such support, professionals may struggle to design interventions that effectively reach underserved populations, thereby contributing to the persistent digital and health divide between those with higher and lower SEP (8). This observation aligns with previous findings that emphasize a disconnect between general eHealth frameworks and the needs of practice. For instance, while existing models are valuable at a conceptual level, they frequently fail to address day-to-day implementation challenges (37). Others have argued that digital health interventions can even exacerbate inequalities when they are not designed with contextual, socio-economic factors in mind (6), highlighting the need for concrete, context-sensitive guidance tailored to underserved populations. Additionally, digital health tools often risk reinforcing stereotypes by framing people with a lower SEP as passive or digitally incapable. This tendency can undermine inclusive design unless explicitly countered through participatory approaches that recognize the diversity and agency within this group (7), something the IeG attempts to address. In line with this, making implicit professional knowledge explicit is critical to improving practical decision-making. And emphasize that surfacing such tacit knowledge supports more responsive, situated design, a core ambition of the IeG (38). The Digital Public Health Framework (39) has highlighted the need for frameworks that consider both system-level ethics and practical utility, yet its emphasis remains at the policy and strategic level. In contrast, tools like the IeG aim to operationalize these principles by offering hands-on, context-specific advice for day-to-day use.

Moreover, gathering feedback from participants underscores the iterative development's essential role in the IeG's evolution. Through a cyclical process of prototyping, testing, analyzing, and refining (24), the guide remains responsive to user feedback and adaptable to emerging challenges. This dynamic approach is crucial for ensuring the IeG meets the field's evolving needs, becoming a more impactful resource for professionals in eHealth development.

## 5 Implications for practice and research

Our study highlighted the necessity of combining scientific and practical knowledge for eHealth professionals. This integration is essential for developing evidence-based eHealth interventions responsive to users' needs. As illustrated by prior research (40) on workplace interventions, connecting research with practical application ensures research remains relevant, actionable, and closely tailored to the practical contexts and specific needs where it is applied. Implementing a similar approach in eHealth can help guarantee that interventions are rooted in solid scientific evidence and are also user-friendly and tailored to address the challenges faced by end-users, thereby improving the effectiveness and impact of these interventions.

Future research should prioritize assessing the long-term impacts of the IeG on eHealth interventions' development and outcomes. Diversifying communication channels, such as instructional videos and interactive workshops, and engaging a wider range of stakeholders could enhance the IeG's reach and adoption. Furthermore, we propose that establishing professional learning communities among eHealth professionals to share practical experiences and scientific research can enrich the collective knowledge base, aiding in developing more effective, user-centered interventions. Continuous refinement, informed by ongoing user feedback (e.g., co-design) and advancements in eHealth technologies, is essential for its sustained relevance. Regular updates are necessary to keep the IeG aligned with the evolving eHealth landscape and to promote inclusive healthcare practices. Expanding implementation and dissemination, possibly using frameworks like RE-AIM (Reach, Effectiveness, Adoption, Implementation, and Maintenance) (41), can enhance the guide's utilization.

### 5.1 Strengths and limitations

The strengths of this study include a mixed-method approach combining quantitative approach with qualitative interviews, using the think-aloud technique to understand the strengths, practical use and areas for improvement of the IeG from the perspective of targeted professional users. The diversity of professionals involved provided a broad evaluation, offering varied viewpoints. However, limitations exist. The selection of participants might have introduced bias, possibly including only professionals who see potential in eHealth for lower SEP. Due to time constraints and professional availability, it was not feasible to involve more than one individual from each eHealth intervention, limiting the breadth of perspectives. Excluding lower SEP individuals from the feedback process, which could have provided critical insights into the guide's accessibility and relevance. Future research should therefore include individuals with lived experience of lower SEP in order to deepen the evaluation and ensure the guide's inclusiveness. Moreover, the potential for socially desirable responses was heightened by the interviewer's dual role as the IeG developer, possibly leading to an overestimation of the guide's. Future evaluations should use log data to objectively assess usage and effectiveness and mitigating potential biases. Finally, evaluating the IeG based on hypothetical scenarios might not fully capture its practical impact. Assessing real-world applications is essential to evaluate its effectiveness and applicability more accurately.

## 6 Conclusion

The IeG emerges as an valuable resource for professionals aiming to develop accessible eHealth interventions for lower SEP individuals. While the guide is appreciated for its practicality and relevance, the feedback indicates areas for improvement, highlighting the significance of continual refinement. As eHealth evolves, it is crucial that the IeG adapts to meet the dynamic needs of professionals and the communities they serve. By promoting a culture of continuous improvement and integrating scientific and practical insights, the IeG stands as a crucial tool in advancing eHealth solutions, ensuring it remains at the forefront of eHealth innovations and applications.

## Data Availability

The original contributions presented in the study are included in the article/Supplementary Material, further inquiries can be directed to the corresponding authors.
