# Supplementary material for: Content evaluation of the inclusive eHealth guide: how to develop interventions for people with a lower socioeconomic position?
Source: Front Digit Health. 2025 Oct 1;7:1528860. doi: 10.3389/fdgth.2025.1528860 (PMC12521427; doi:10.3389/fdgth.2025.1528860)
Supplement: Supplementary file 1 [file Datasheet1.docx]

Supplementary Material 1

## Supplementary Figures

# Supplementary Figure 1. Screenshots of the Inclusive eHealth Guide

**Home page Guide**


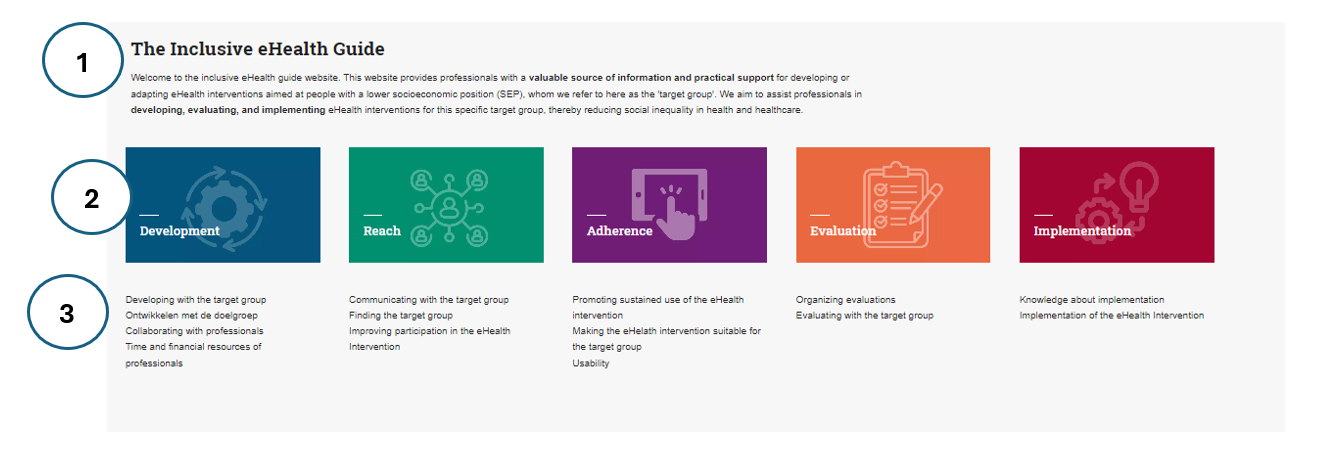


**Example of recommendations 'Works' and 'Doesn't Work'**


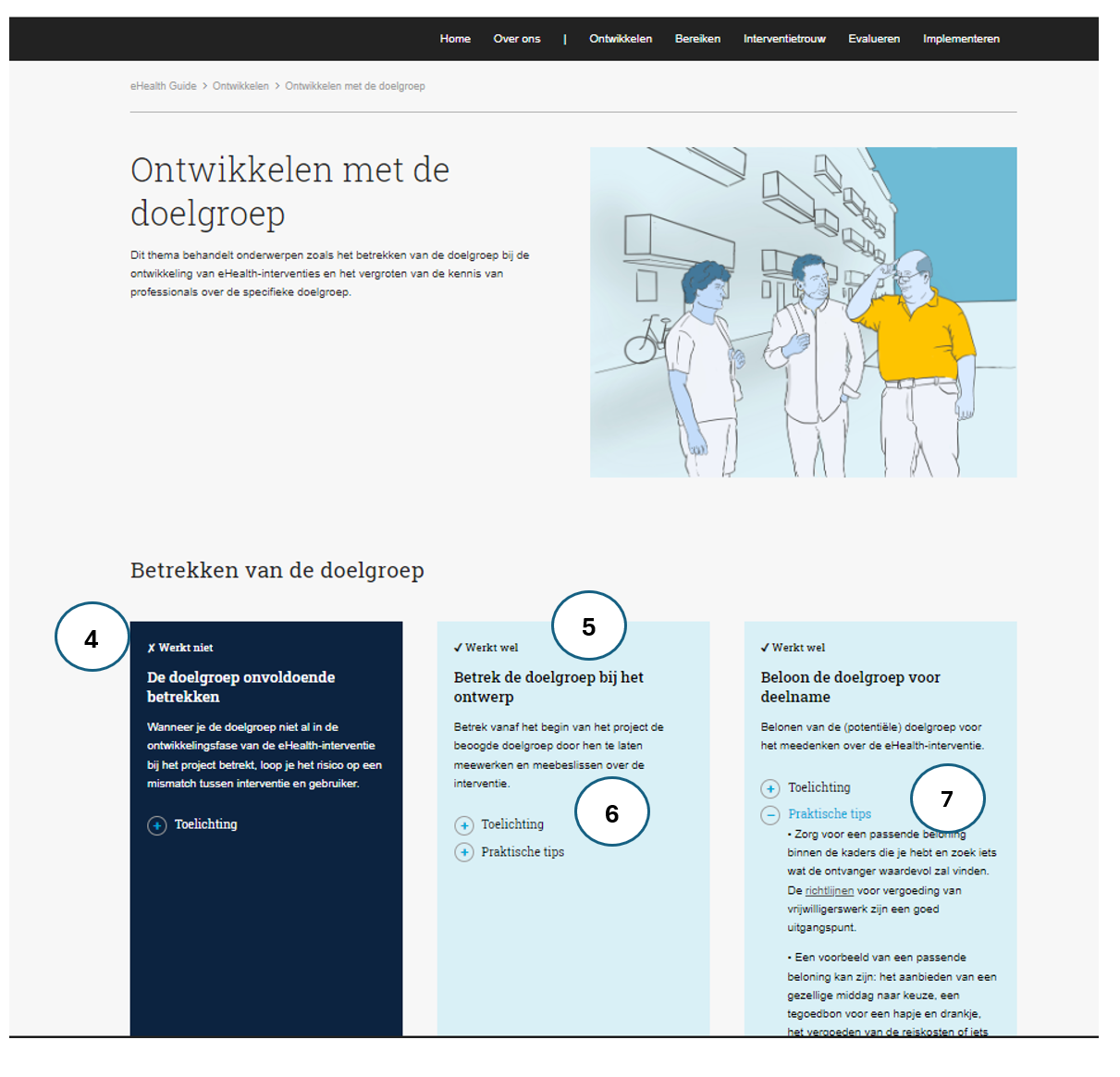


Home page

1. Starting Scheme: Upon accessing the home page, users are immediately presented with a scheme that offers a comprehensive overview of the guide's contents. This provides a quick understanding of what's available and helps them identify the most relevant sections.
2. Phases: This part of the guide details the various phases of eHealth interventions, providing users with easy navigation to the specific phase they are interested in exploring further.
3. Themes: Lists and describes themes related to the different phases of eHealth interventions, offering users insights into the topical structure of the guide.
4. What Does Not Work: Highlights recommendations known to serve as barriers, cautioning users against common pitfalls in eHealth interventions.
5. What Does Work: Features facilitative recommendations that have been identified as useful, supporting the successful implementation of eHealth interventions.
6. Explanation: Offers brief clarifications on why certain recommendations are marked as 'Does Not Work' and why others are endorsed as 'Does Work.'
7. Practical Tips: Provides users with directly applicable information, including practical tips, illustrative examples, external tools, resources, and references to foundational literature.

## Supplementary Tables

## Supplementary Table 1. Overview of eHealth interventions by Type and Demographic

| Type of eHealth Intervention (N=8) | Target group | Province(s) |
| --- | --- | --- |
| Multiple lifestyle (n=4) | Hospital employees, women of childbearing age and their partners, and residents aged 45-67 in specific neighborhoods, and older adults with Type 2 diabetes | South Holland and North Holland (n=6) |
| Physical Activity (n=2) | General population and women with prior hypertensive pregnancy | South Holland and North Holland (n=1) |
| Sleep (n=1) | General population | North Holland (n=2) |
| Smoking Cessation (n=1) | Smokers with a lower SEP | Utrecht (n=1) |

# Supplementary Table 2. User types and Timing for utilizing the IeG

| Theme | Theme mentions (n) | Participants (n) | Description | Quote |
| --- | --- | --- | --- | --- |
| General |  |  |  |  |
| Category: Type of guide users |  |  |  |  |
| Diverse group | 7 | 6 | Diverse professionals | *Say, the executing professionals who are involved in the actual implementation.* [P2] |
| eHealth developers | 7 | 6 | Digital health creators | *No, I would say developers of eHealth interventions …* [P2] |
| Health professionals | 3 | 5 | Healthcare providers | *But also for people who are going to provide support. In such an intervention, so that can also be a healthcare professional.* [P2] |
| Project managers | 2 | 2 | Leaders of the projects | *... but also for the project managers who are managing it for all the people, program managers who are developing these things.* [P5] |
| Researchers | 6 | 6 | Researchers who are involved in the different process | *Those who carry it out, but I can imagine that developing is just very important for the developers. But also definitely for researchers.* [P12] |
| Category: Evidence type |  |  |  |  |
| Scientific Evidence | 15 | 11 | Balancing scientific evidence and practical insights in recommendations | *I think the scientific basis is always very good because that's just the strongest form of knowledge.* [P26] |
| Combination of Scientific and Experiential Knowledge | 6 | 5 | Merging scientific research with experiential knowledge for robust guidance | *… Participants' experiences or patients' experiences. Or whatever if that also falls under scientific evidence then not just effectiveness or whatever. But I think the most important is the combination of experts and the target group itself.* [P3] |
| Experiential Knowledge | 7 | 6 | Valuing practical experience and best practices in the field. | *No, because this is all very practical and some things may not be documented. Somewhere of course, but that for example, I don't know how many studies there are about. We had too little money and then the implementation flopped. Yes, they are there of course, but even if it's not necessarily in the literature like that, then it's still an important tip that you just don't have enough money, right? Not everything is I think necessarily research. Yes, maybe it's based somewhere or something.* [P4] |
| Category: Timing use of the guide |  |  |  |  |
| At the Beginning | 7 | 7 | Use the guide before starting projects for a comprehensive overview | *Yes really before you write the grant, yes before you even have a plan about the intervention really at the beginning. I think it's good as background information and then as a guideline in the process.* [P12] |
| Different Moments | 5 | 5 | Refer to the guide at various project stages for continuous learning | *Well, I would also like to look at it now. We are now in the implementation phase with [name of intervention] and also evaluation, so I would definitely want to look at it again to see if there are things we can learn from it.* [P2] |
| Writing Proposal | 2 | 2 | Utilize the guide early in proposal development and planning phases | *Oh, when you make the research proposal, or at least the setup of the development plan.* [P3] |

n theme mentions = total number of times the theme was mentioned

n participants = number of unique participants who mentioned the theme.
