# Supplementary material for: Content evaluation of the inclusive eHealth guide: how to develop interventions for people with a lower socioeconomic position?
Source: Front Digit Health. 2025 Oct 1;7:1528860. doi: 10.3389/fdgth.2025.1528860 (PMC12521427; doi:10.3389/fdgth.2025.1528860)
Supplement: Supplementary file 2 [file Datasheet2.docx]

Supplementary Material 2

## Supplementary Protocol 1. Interview Protocol: Evaluation of the Inclusive eHealth Guide

**Questionnaire Structure:**

Part 1: Background Information

**Part 1a: Background Information**

*This section is to be filled out digitally by the participant prior to the interview conversation. If this is not done, the questions will be asked during the interview.*

1. What is your current position?
2. What is your gender?
3. Do you have experience in conducting research in the field of lifestyle interventions? If so, how long have you had this experience?
4. How long have you been involved with eHealth?
5. Do you professionally work on improving the health of people with a low socioeconomic position (SEP)?

- Yes
- A little
- No

1. In what way are you involved with people with a low SEP through your work? Multiple answers possible.

- Developing or adapting (eHealth) interventions
- Applying (eHealth) interventions to people with low SEP
- Policy development
- Scientific research
- Working in healthcare
- Other: __________

*Source: Wentzel et. 2016; Developing Accessibility Design Guidelines for Wearables: Accessibility Standards for Multimodal Wearable Devices*

1. Do you have experience in designing eHealth for people with low socioeconomic status, such as people with a lower educational level or lower income, or with 'design for all'? If so, could you describe this experience?
2. What documents, websites, guidelines, or guidelines have you used?
3. What guidelines for developing for people with low SEP are you aware of?
4. Have you used them?
5. What are your reasons for using them?
6. How do you keep yourself informed about developments in the field of eHealth and low SEP?

*Source: Balbach E. D. (1999). Using case studies to do program evaluation. Sacramento CA: California Department of Health Services 139-155.*

**Part 1b: Background Information**

*These questions are asked during the first part of the interview conversation.*

1. How long ago did you get involved in the intervention?
2. What is/was the goal of the eHealth intervention?
3. For which audience is or was the eHealth intervention intended?
4. To what extent did you consider special groups such as people with lower SEP during development/reach/evaluation/implementation, ..?

- Follow-up question:
  - Option 1: Why did you consider this target group?
  - Option 2: Why did you not consider this target group?

Part 2: Evaluating the Guidelines

Part 2 consists of several steps:

- Step 1: Briefly explain the different phases.
- Step 2: Ask the participant to read the scenario.
- Step 3: Check if the objective matches the participant's thoughts.
- Step 4: Have the participant view the website.
- Step 5: Ask the participant questions.

**Step 1: Briefly Explain the Different Phases.**

|  | Phase | Explanation |
| --- | --- | --- |
|  | Developing an eHealth intervention | This includes designing, developing, and testing an eHealth application to address health problems. |
|  | Reaching people with a low SEP | This involves trying to reach the target audience of the eHealth intervention, known to have possibly less access to technology and less health-related knowledge and skills. |
|  | Promoting intervention adherence  Implementing an eHealth intervention: | This refers to encouraging users' engagement and following the digital health intervention. |
|  | Evaluating an eHealth intervention | This involves examining whether the eHealth application is effective and meets the set goals and expectations. |
|  | Implementing an eHealth intervention | This is the process of implementing the eHealth application in healthcare practice and making it available to the target audience. |

**Step 2: Scenario.**

Situation: You are currently developing [name] intervention for a broader group, including people with a low SEP.

| Scenario - Researcher (Development)  Situation: You are currently working on developing the [name] intervention for a broader audience, including those with a low socioeconomic position (SEP).  Website: Imagine that there is a website designed to support professionals like you in the development, implementation, and evaluation of your eHealth intervention for individuals with a low socioeconomic position (SEP). This platform aims to help you reach this group more effectively and improve intervention adherence among them.  Action: You are curious about the information available and decide to explore this resource.  Goal Setting: What kind of information are you looking for or are you in need of |
| --- |

**Step 4: Have the Participant View the Website.**

**Instructions:**

- Please click on ... to go to the homepage of the guidelines website.
- When you click on [...], you will arrive at the page about [...]. Take your time to explore this page. I will ask you questions about it shortly.
- Please read or scan the information at your leisure and feel free to point out anything that catches your attention. Would you be willing to share your thoughts aloud?
- Have you seen the recommendations and practical tips? If you haven't read them yet, would you like to go through them?

**Step 5: Ask the participant questions.**

*You have just reviewed the guidelines. I would like to ask you a few questions regarding the guidelines. I kindly request you to assign a score for each question that best reflects your opinion about the guidelines. You can also refer back to the guidelines if you need to review the information again.*

| **Question** | **Response Options** | **Note** |
| --- | --- | --- |
| 1. To what extent do you find that the guidelines contain the information you would need to develop an eHealth intervention for people with low SEP? | - - - - - - +/- - + - ++ | If applicable, ask question 2. |
| 2. To what extent do you find that the guidelines contain the information you would need to reach people with low SEP? | ,, | If applicable, ask question 3. |
| 3. To what extent do you find that the guidelines provide sufficient information to promote long-term use and adherence of the eHealth intervention for people with low SEP? | ,, | If applicable, ask question 4. |
| 4. To what extent do you find that the guidelines provide sufficient information to evaluate an eHealth intervention for people with low SEP? | ,, | If applicable, ask question 5. |
| 5. To what extent do you find that the guidelines contain the information you would need to implement an eHealth intervention for people with low SEP? | ,, |  |
| 6. What are your thoughts on the included recommendations (inhibiting and facilitating factors)? | ,, | Follow-up questions:  - Can you explain further why?  - Which recommendations did you find most and least appealing, and why?  - Show some examples. |
| 7. To what extent do you find the practical information (examples and tips) useful? | ,, |  |
| 8. To what extent do you find that the recommendations are clearly and concisely described? | ,, |  |
| 9. To what extent do you find that the information density of the recommendations is sufficient? | ,, |  |
| 10. To what extent do you find that the recommendations contain enough information for you to start working on your own? | ,, |  |
| 11. To what extent do you find that the guidelines provide enough information to make the eHealth intervention [project name] suitable for people with low SEP? | ,, |  |
| 12. Do you believe it is necessary for the recommendations to be based on scientific evidence? | ,, | Follow-up question: Or can experiential knowledge and 'best practices' also be useful? |

Part 3: Final Questions

1. Can you imagine using these guidelines?
   - Please explain your answer.
2. For whom do you think these guidelines would be useful?
   - Please explain your answer.
3. At what moment would you want to use the guidelines?
   - Please explain your answer.
4. Lastly, imagine if these guidelines had been available when you were working on developing your eHealth intervention [specify the name]. Which aspects of the guidelines would you have found most useful, and why?
